# Non-Mammalian Nuclear Receptors: From Evolution to Human Disease

**DOI:** 10.11131/2018/101366

**Published:** 2018

**Authors:** Chris R. Gissendanner, William S. Baldwin, Marcel J. M. Schaaf

**Affiliations:** 1 Biology Program, School of Sciences, University of Louisiana Monroe, Monroe, LA 71209, USA; 2 Biological Sciences, Clemson University, Clemson SC 29634, USA; 3 Environmental Toxicology Program, Clemson University, Clemson SC 29634, USA; 4 Institute of Biology (IBL), Leiden University, Leiden, The Netherlands

In 1964, Ulrich Clever published a landmark paper on the actions of 20-hydroxyecdysone (20E), the hormone that regulates molting and metamorphosis in insects. Based on the puffing activity of *Chironomus tentans* salivary gland polytene chromosomes, Clever was able to establish a pattern of gene activation in response to 20E [1]. A decade later, Michael Ashburner, utilizing *Drosophilamelanogaster*salivary gland polytene chromosomes, established a formalized model (the “Ashburner Model”) where 20E, bound to its receptor, activates a set of primary (“early”) target genes. The products of these genes, in turn, repress their own expression, and activate the transcription of secondary (“late”) target genes that control metamorphosis [2]. Subsequently, it was shown that the receptor for 20E, EcR, was an insect member of the nuclear receptor superfamily, and EcR, along with its heterodimer partner Usp (homolog of RXR), bind 20E and activate a set of early target genes at the onset of metamorphosis [3,4]. Additionally, many of the early gene products were also nuclear receptors that regulated the transcription of the secondary late genes [4]. Thus, the fruit fly as an invertebrate model system for studying nuclear receptor signaling was established. The regulation of metamorphosis, in association with powerful fruit fly genetics, became an important system for deciphering the mechanisms of nuclear receptor action, and subsequent studies demonstrated the utility that invertebrate species such as the fruit fly possess in understanding nuclear receptor function in the context of a whole organism.

Many of the different nuclear receptor (NR) superfamily groups are conserved throughout the Metazoa. Therefore, studies of nuclear receptors in non-mammalian species, including genetic model organisms such as *Drosophila* and zebrafish (*Danio rerio*) and environmentally relevant species such as *Fundulus* and *Daphnia*, provide an essential complement to mammalian NR research. Such studies can provide insights into deep, conserved aspects of NR signaling, and ecologically adapted functional roles [5]. Additionally, such studies have yielded many unexpected findings. For example, the genome of *Caenorhabditis elegans*, a free-living soil nematode, encodes greater than 280 nuclear receptors, due to an apparent expansion and diversification of the HNF4 group [6]. NRs evolved from a likely ancestral fatty acid receptor to a diverse group of receptors that recognize multiple ligands, or can be ligand-independent transcription factors due to subtle modification of the internal cavity [7]. Non-mammalian vertebrate organisms have also provided important insights. Zebrafish has an array of genetic tools at its disposal and is especially well-suited for studies related to embryonic development and organogenesis. It has been particularly useful in probing various aspects of steroid NR signaling, as well as the evolution of promiscuous receptors [8,9]. Finally, comparative analyses of NRs in non-mammalian species have been essential in revealing the evolutionary history of this transcription factor superfamily [10].

Despite the many important discoveries that have come from nuclear receptor research in non-mammalian systems, these organisms are still largely underutilized. In this special issue of *Nuclear Receptor Research,* we present five articles that further demonstrate the insights that can be gained by investigating nuclear receptors in non-mammalian systems.

In Bodofsky *et al*, [11] the authors take a broad view across three model organisms- *Drosophila, C. elegans,* and mouse- and assess not only the functional conservation of NRs during development but also where NRs have been exapted, or repurposed for different functions in different species. They conclude that exaptation is emphasized over functional conservation with respect to development in these species (http://www.agialpress.com/journals/nurr/2017/101305/).

In Praslicka *et al*, [12] the authors utilize ChiP-seq to identify binding sites and target genes of the *C. elegans* NR4A ortholog, NHR-6. Prior work had demonstrated that NHR-6 regulates cell proliferation and cell differentiation during organogenesis, and the authors found evidence for a complex network of genes regulated by NHR-6, including genes with cell cycle, chromatin modification, cell signaling, and metabolic functions (http://www.agialpress.com/journals/nurr/2017/101288/).

A review article by Marcel J.M. Schaaf [13] provides an overview of fifteen years of steroid receptor research in zebrafish. The review illustrates the importance of zebrafish in studying the effects of endocrine disrupting chemicals, and provides a comprehensive of overview of zebrafish ERs, PR, AR GR, and MR. Current and future trends in zebrafish NR research, such as its use in phenotypic screening of novel steroid drugs, are highlighted (http://www.agialpress.com/journals/nurr/2017/101286/).

The Baldwin *et al* article [14] describes the phylogenetic analysis and annotation of nuclear receptor genes in *Fundulus heteroclitus*(mummichog; Atlantic killifish). This species is an important field model for environmental research due to its ability to respond to and withstand variable environmental conditions. The authors describe the conservation, divergence, expansion, and absence of specific NRs, including the co-expansion of the opposing NRs Reverb-ROR and RAR/RXR-COUPTF, and the absence of the common heterodimeric partner, RXR*α* (http://www.agialpress.com/journals/nurr/2017/101285/).

Finally, the article by Holzer and Laudet [15] describes the discovery of two additional thyroid hormone receptor (TR) genes in the sea lamprey, *Petromyzon marinus.* This result is very interesting since *P. marinus* is an agnathan, a jawless vertebrate, and is an important species for understanding vertebrate evolution. This finding adds an intriguing piece to the puzzle of TR evolution since thyroid hormone signaling is thought to have first appeared in agnathans (http://www.agialpress.com/journals/nurr/2017/101287/).
